# Testing the Club Convergence Dynamics of the COVID-19 Vaccination Rates Across the OECD Countries

**DOI:** 10.3389/fpubh.2022.872561

**Published:** 2022-05-06

**Authors:** Weibin Xu, Chi Keung Marco Lau, Dongna Zhang, Oladoke Oke

**Affiliations:** ^1^Zhejiang Yuexiu University of Foreign Languages, Shaoxing, China; ^2^Department of Economics and Finance, Hang Seng University of Hong Kong, Hong Kong, Hong Kong SAR, China; ^3^Teesside University Business School, Middlesbrough, United Kingdom; ^4^Huddersfield Business School, University of Huddersfield, Huddersfield, United Kingdom

**Keywords:** COVID-19 pandemic, distribution of vaccines, vaccination rates, economic growth model, cluster convergence analysis, OECD countries

## Abstract

Vaccines are essential to create a more resilient economic growth model. Ending the COVID-19 pandemic requires a more coordinated, effective, and equitable distribution of vaccines across the countries. Therefore, governments are in a race to increase the vaccination rates of the population. Given this backdrop, this paper focuses on the daily vaccinations per million data from March 1, 2021, to October 15, 2021, in 37 Organization for Economic Co-operation and Development (OECD) countries and examines the stochastic properties of the vaccination rates. We adopt the club convergence econometric methodology to investigate the club convergence paths of COVID-19 vaccination rates in OECD regions. The results indicate a significant convergence of the vaccination rates in seven clubs across 30 OECD countries. Moreover, there are seven OECD countries demonstrate non-convergent characteristics, which raises questions about ineffective vaccine balance. In addition, the paper also discusses the potential implications for the post-COVID-19 era.

## Introduction

The race for vaccine inoculation exemplified today's glaring global health and economic inequalities: countries' capacity to immunize their populations has been proportional to their relative cost (1). The world economy has now seen vaccines deployed as a geopolitical weapon. One significant concept that has emerged recently is the concept of vaccination imbalance. This issue is a phenomenon that describes the competition between dozens of “rich” countries to obtain deals with pharmaceutical corporations to assure vaccines for their populations; therefore, limiting the supply of vaccines for less fortunate countries that lack the same bargaining power (2). A vaccine imbalance will obstruct the global economy's inclusive recovery. No region can be considered safe unless all areas are considered safe. The rapid spread of COVID-19 variations globally necessitates concerted action among countries to ensure that global vaccination is timely, equitable, and inclusive (3).

However, reactivating the economy is impossible until the pandemic is brought under control. Simultaneously, when the economy recovers, the probability of transmission increases. Therefore, complementarity between health and economic policies (fiscal, production, and social) is critical for containing the epidemic and starting the economic reactivation process. Although the concept of health-economic interconnectedness is not new, the COVID 19 pandemic has amply proved this relationship. This interconnectedness is a structural problem for governments. It necessitates the adoption of long-term policies that foster virtuous health and growth dynamics. Sustainable economic growth is critical for the health and wellbeing of individuals. Simultaneously, safeguarding and promoting the population's health should serve as the foundation for a long-term growth and development strategy.

Although vaccine implementation is patchy it is gaining momentum. Emerging statistics demonstrate that economic activities have increased and partially adjusted to pandemic constraints in many areas of the global economy (4). Government stimulus is projected to significantly stimulate economic activity. However, possibilities for sustained growth vary significantly by country and sector. They are contingent on vaccinations being distributed fairly to all countries.

Countries that have been proactive in vaccinating their citizens against the COVID-19 and successfully controlling infections through good public health policies have seen a faster recovery of their economies. The global job market is improving, including services, such as tourism. However, while vaccination rates increase in many developed economies, they fall behind in developing and emerging economies. Even in developed countries, there is considerable disparity in the vaccine rollout. In the OECD countries, for example, the share of people who have been fully vaccinated reached over 90% in Portugal and Chile. They are only 50% in Slovakia and <60% in Poland. This discrepancy underscores the importance of all countries and areas benefiting from a stronger, more rapid global economic recovery which can only be achieved if there is balance in vaccine rollout and administration.

Emergencies involving public health (the COVID-19 pandemic) necessitate the rapid, efficient, and large-scale distribution of crucial medical interventions. Paramount among these interventions are vaccines, drugs, and therapeutics (5). Their distribution is complicated and necessitates careful planning and coordination from global government, private institutions, and multi-agency departments. The OECD (2) suggests that although there has been some progress in the vaccination rollout against COVID-19, the emergence of variations threatens the speed of economic recovery, demonstrates the fragility of the World's prosperity, and emphasizes the importance of developing a more resilient and sustainable growth and development paradigm. The OECD (3) also submits that the critical first step in ascending the path of sustainable growth post-COVID-19 pandemic is to improve the global immunization program's uptake, efficiency, effectiveness, coordination, and equity.

However, the uptake and dispensing of vaccines quickly and efficiently is a top shared international goal among the OECD community during public health emergencies. Vaccinating a large percentage of the population is critical in order to effectively contain the pandemic and avert further deaths, however due to the novelty of the virus there is still a lot of regional learning gap that impedes the formation of formidable regional alliance to combat the pandemic. Indeed there are several ways the effectiveness of a vaccine regime can be approached but earlier convergence hypothesis (6) suggests that policies are better channeled to converging clubs in order to magnify policy effectiveness (7, 8). Additionally, the multifaceted nature of dispensing and local coordination to manage the COVID 19 outbreak has been paramount. Importantly, lack of cohesion and unison in regional policy thrust has been evident as countries have taken largely individualistic approaches to addressing the pandemic other than united arsenal of policy actions. These problems point to the fact that a lack of convergent or divergent view of the problem might have provided a guided approach to solving the pandemic issues in the OECD. With the wide disparity of vaccination intent globally^1^, there is a clear gap in understanding the convergence of efficient vaccination uptake (2).

This paper examines the convergence of vaccination among OECD countries in response to the COVID-19 pandemic and investigates unifying factors among similar clubs. We adopt the club convergence econometric methodology introduced by Phillips and Sul (9, 10) to investigate the club convergence paths of COVID-19 vaccination rates in OECD regions. It is particularly instructive to study the convergence of vaccination rates to isolate policies that lead to a more efficient vaccination rate within the OECD. Health policy choices might undoubtedly influence long-term economic development. Still, such concerns are rarely explicitly included in vaccination analysis. They are also likely to fall outside of the planning horizon of the policymakers, especially because of the seasonality effects of pandemics, like the COVID-19.

The remainder of the paper proceeds as follows—section Related Literature reviews relevant literature. Section Econometric Methodology specifies the methodology. Section Empirical Results discusses the results. Section Conclusion concludes.

## Related Literature

Studies have shown the importance of vaccines and vaccination programs for economic development (11–14). However, the literature suggests that convergence studies on health expenditures (vaccine and vaccinations inclusive) are necessary to analyze countrie's differentials in health outcomes.

Neoclassical growth theory is used to explain economic convergence. According to the hypothesis, countries with similar desires and technology will eventually reach a steady-state level of wealth per capita. Developing countries grow faster than rich countries, eventually catching up. Since health spending rises as income rises and health spending may also converge when incomes rise in different countries, it is assumed that we can find similarities in health policies of countries in the same club convergence. The literature on health expenditure convergence between countries has been motivated by this assumption.

Furthermore, the literature has focused broadly on health expenditure convergence (15–19). However, Islam et al. (20) and Zhai et al. (21) argue that evidence from the current COVID-19 pandemic indicates that illness severity and case fatality rates vary significantly around the globe and thus a greater knowledge of the COVID-19's epidemiological characteristics, particularly why certain populations are more susceptible, might aid in the successful control of this pandemic. Against this backdrop, this paper investigates vaccination convergence of COVID-19 among the OECD nations by accentuating policies that promote vaccination efficiency within the co-operation.

Lanzavecchia et al. (22) suggest that Chile, an OECD country, astonished the globe with a high COVID-19 immunization rate. Despite this, incidents hit a new peak in April 2021, prompting the country to declare a state of emergency. There are numerous, complex, and interwoven reasons behind this. The resurgence appears to be caused by a false sense of security at the start of vaccination, the introduction of novel, more transmissible variations, too early relaxation of non-pharmacological precautions before herd immunity threshold. The political background and socioeconomic inequality in Chile, on the other hand, play a significant role and are more difficult to measure and compare with other nations. Finally, the Chilean example serves as a reminder not to rely just on vaccination rates to limit the pandemic but also to keep some of the earlier non-pharmaceutical tactics in place.

Similarly, Chen (23) submits that vaccination rates and efficiency vary widely. As of May 24, 2021, the fraction of the population fully vaccinated against COVID-19 in some nations had surpassed 50%. The uptake is as low as <1% in many other countries. Currently, the literature attempting to explain the wide global disparity in COVID-19 vaccination efficiency is still budding. None approaches the problem with a club convergence methodology. However, Chen (23) investigated the variations in the uptake of COVID-19 vaccinations in attacking the spread of the virus. As nations in the globe have yet to achieve herd immunity, several countries were chosen as test cases based on the following criteria: more than 60 vaccination doses per 100 individuals and a population of more than one million people. Israel, the United Arab Emirates, Chile, the United Kingdom, the United States, and Qatar were chosen as the seven countries. The study contends that vaccination has a significant impact on lowering illness rates in all nations.

Furthermore, the findings suggest that the infection rate after immunization, on the other hand, showed two distinct patterns. One is an L-shaped trend, while the other is an inverted U-shaped trend. When the vaccination rate reaches 1.46–50.91 doses per 100 individuals in nations with an inverted U-shaped pattern, the infection rate declines.

For brevity, we find that many more studies (24–26) that towed this line of philosophy generally suggest that vaccination uptakes are useful for stemming down infection rates; however, these studies fail to extant juxtapose the health policies that have proved successful in the face of individual countries' vaccination roll out and to isolate similarities in policies that promote vaccination efficiency. One econometric way to solve this problem is to analyze the phenomenon via the lenses of club convergence (27, 28).

Kong et al. (29) expatiated that the idea of relative convergence, which states that the ratio of two-time series must eventually converge to unity, explains why series exhibit converging behavior when they share typically divergent stochastic or deterministic trend components. This form of relative convergence may not always hold when series exhibit similar time decay patterns as evaluated by evaporating rather than diverging trend behavior. To account for convergent behavior in panel data that do not contain stochastic or divergent deterministic trends, the study developed the concept of weak convergence, which refers to the process through which cross-section variation in the panel diminishes with time. The research formalizes this concept and presents a straightforward linear trend regression test for the null hypothesis of non-convergence. The test's asymptotic qualities are developed under general regularity constraints and using a variety of data generation procedures. Simulations demonstrate that the test has an acceptable level of size control and discriminatory power. The approach is used to investigate whether the idiosyncratic components of 46 disaggregated personal consumption expenditure price inflation items converge over time, with substantial evidence of poor convergence in these data. In a second application, the method is used to determine if experimental data from ultimatum games converge over subsequent rounds, with the method again demonstrating weak convergence. A third application examines the convergence and divergence of unemployment data for the United States of America from 2001 to 2016.

Karakaya et al. (30) also examine the primary driving forces behind the EU's material use in the situation of heterogeneity across member states, with a particular emphasis on the comparison of domestic material consumption (DMC) and material footprint (MF). To accomplish this, they first divide nations into numerous clubs based on the time-varying behavior of two material use indicators and then examine the degree of heterogeneity among EU member states. Second, they apply the logarithmic mean Divisia index (LMDI) decomposition analysis to determine the primary driving reasons behind DMC and MF changes and examine the material use features unique to each club. The empirical findings indicate that the EU member states do not converge to a single steady-state level and that both indicators exhibit multiple equilibria. Since the number of clubs per capita DMC was bigger than the number of clubs per capita MF, the income impact had the highest effect on material consumption, and next on the population effect. The authors submit that the number of effects varied greatly between clubs.

Ulucak and Apergis (31) examined the convergence of the per capita ecological footprint using annual data for the EU member states from 1961 to 2013. The authors employed the club clustering technique, and the empirical evidence demonstrates the existence of certain convergent clubs. Their empirical findings shed light on the disparities in environmental quality and the EU member's awareness tactics each club must employ. They identify that ignorance about the ecological footprint's importance in the health of our ecosystems has the potential to have unintended and detrimental consequences for the future. They argue that their findings have major policy implications for energy and environmental regulators and that nations must adopt innovative techniques that significantly contribute to a sustainable growth process while maintaining high qualitative environmental standards.

Following Barro and Sala-i-Martin (32), convergence studies have received copious attention in a variety of fields of macroeconomic theory. When employing various empirical techniques, there are numerous consequences for convergence, including time series, cross-sectional, and panel data. Most of the literature utilizes that convergence regression through an economic growth equation within Solow's (33) neoclassical growth theory.

These studies vary in terms of the variables they examine, ranging from ecological footprints, commodity prices, public spending on health, defense, and education, to fiscal and monetary variables, foreign trade, tourism, and energy consumption (19, 30, 31, 34–37). However, studies focusing on vaccination convergence are almost non-existent to the best of our findings, although the literature is replete with healthcare expenditure convergence which forms the basis for the contribution of this paper. Indeed, the COVID-19 pandemic now urgently warrants the search for new knowledge that can take the World out of the woods via health policies that improve or sustain the efficiency of vaccination uptake.

## Econometric Methodology

To examine the club convergence paths of COVID-19 vaccination rates in OECD regions, we employ the club convergence econometric methodology introduced by Phillips and Sul (9, 10). We assume the variable represents the vaccination rates Z_itWhere i refers to the number of OECD countries with i= 1,2, …, N; t refers to the period with t = 1,2, …, T. Under a nonlinear time-varying factor model framework, Z_it can be decomposed into a common component and a time-varying idiosyncratic element as follows:


(1) $$\begin{array}{r} {\text{Z}}_{\text{it}}\;=\;\beta_{\text{it}}\omega_{\text{t }}\text{for all i and t} \end{array}$$

where $\beta_{it}=\frac{\beta_{i}}{L\left(t\right)t^{\alpha }}\;$represents the time-varying common element, *L*(*t*) = log(*t*+1) and ω_*it*_ represents a time-varying idiosyncratic element. To measure the transition path loading coefficient β_*it*_ regarding the panel average, Phillips and Sul (9) propose a relative transition measure *p*_*it*_ as follows:


(2) $$\begin{array}{r} {\text{p}}_{\text{it}}\;=\;\frac{{\text{Z}}_{\text{it}}}{\frac{1}{\text{N}}\sum_{\text{i }=\;1}^{\text{N}}{\text{Z}}_{\text{it}}}\;=\;\frac{\beta_{\text{it}}\omega_{\text{t}}}{\frac{1}{\text{N}}\sum_{\text{i }=\;1}^{\text{N}}\beta_{\text{it}}\omega_{\text{t}}}\;=\;\frac{\beta_{\text{it}}}{\frac{1}{\text{N}}\sum_{\text{i }=\;1}^{\text{N}}\beta_{\text{it}}} \end{array}$$

The trend element Ẑ_*it*_ is then extracted from *Z*_*it*_ to be used in the computation of the transition path ${\hat{p}}_{it}$, as:


(3) $$\begin{array}{r} {\hat{p}}_{it}\;=\;\frac{{Ẑ}_{it}}{\frac{1}{\text{N}}\sum_{i\;=\;1}^{N}{Ẑ}_{it}} \end{array}$$

The null hypothesis of convergence is tested in the below estimation equation:


(4) $$\begin{array}{r} \mathrm{Log}\left(\frac{{\text{P}}_{1}}{{\text{P}}_{\text{t}}}\;\right)\;-\;2log\text{L}\left(\text{t}\right)\;=\;\hat{\text{m}}\log\text{t }+\;\hat{\text{n}}\;+\;{\hat{\text{v}}}_{\text{t}} \end{array}$$

where $P_{t}=\frac{1}{N}\sum_{i=1}^{N}{\left({\hat{p}}_{it}-1\right)}^{2}$, *L*(*t*) = log(*t*+1). In particular, $\hat{m}=2\hat{\alpha }$ and $\hat{\alpha }$ is the ordinary least squares estimate of α.

Based upon the above empirical specification, the convergence behavior can be tested via log t regressions with the null hypothesis H_0 and the alternative hypothesis H_1for all i:


(5) $$\begin{array}{r} {\text{H}}_{0}:\delta_{\text{i}}\;=\;\delta ,\;\alpha \geq 0. \end{array}$$

(6) $$\begin{array}{r} {\text{H}}_{1}:\delta_{\text{i}}\;\neq \;\delta \text{ or }\alpha <0. \end{array}$$

Through the utilization of the one-sided t-statistic t_m_, the null hypothesis of convergence cannot be rejected if t_m_>−1.65.

However, it is worth noting that the rejection of the null hypothesis across the panel does not inform the existence of club convergence. To further investigate the characteristics of the convergence dynamics, Phillips and Sul (9, 10) developed an econometric procedure to identify club convergence and clustering features summarized in four steps. In the first step (ordering), the N countries in the panel series are ordered based on the final observation. In the second step (core group formation): the convergence t-statistic *t*_*j*_ is calculated for the log t regressions according to the j highest members from the panel with j should be in the range of 2 to N. The core group number is determined by the maximum value of *t*_*j*_ with *t*_*j*_>−1.65. Then, in the third step (club membership): countries for membership in the core group are chosen by selecting each country individually. It is important to ensure that the convergence criterion rule is satisfied throughout the process.

Finally, in the last step (recursion and stopping), a complement group includes the countries not chosen in the core group from the third step. A log t regression is used to test the convergence behavior of the complement group. A second convergence club is formed if these countries demonstrate a convergence pattern. Alternatively, the first step to the third step is repeated to uncover possible sub-convergence groups. As a result, the vaccination rates of the remaining countries are divergent if no core group is formed as in the second step.

## Epirical Results

### Data Description

We collect the vaccination data for OECD member countries from the Coronavirus (COVID-19) international vaccination database constructed by Our World in Data provided by Mathieu et al. (38). The Our World in Data COVID-19 vaccination database adopts the up-to-date statistics from government officials and health departments across the globe.^2^

The dataset consists of 37 OECD member countries from March 1, 2021, to October 15, 2021.^3^ The vaccination rate is measured as daily vaccinations per million of the population across the list of sample countries. Table 1 outlines the country ID and code for each OECD country included in our sample. As more than 1 year just passed since the first dose of the COVID-19 vaccine in December 2020 was administered, a wave of vaccination race has initiated. It has been ongoing worldwide to end the pandemic of COVID-19. The UK administered the first fully tested vaccination manufactured by Pfizer/BioNTech at the end of 2020. The strategic plan to roll out the vaccine differs at regional, national, and international levels. For example, some regions have decided to administer the vaccine based on the vulnerability of residents. In contrast, other areas have out the rollout speed of the vaccination as a priority.

**Table 1:** Sample of the OECD members.

**Country ID**	**Country**	**Country code**	**Country ID**	**Country**	**Country code**
1	Australia	AUS	20	Latvia	LVA
2	Austria	AUT	21	Lithuania	LTU
3	Belgium	BEL	22	Luxembourg	LUX
4	Canada	CAN	23	Mexico	MEX
5	Chile	CHL	24	Netherlands	NLD
6	Colombia	COL	25	New Zealand	NZL
7	Costa Rica	CRI	26	Norway	NOR
8	Czechia	CZE	27	Poland	POL
9	Denmark	DNK	28	Portugal	PRT
10	Estonia	EST	29	Slovakia	SVK
11	Finland	FIN	30	Slovenia	SVN
12	France	FRA	31	South Korea	KOR
13	Germany	DEU	32	Spain	ESP
14	Greece	GRC	33	Sweden	SWE
15	Iceland	ISL	34	Switzerland	CHE
16	Ireland	IRL	35	Turkey	TUR
17	Israel	ISR	36	United Kingdom	GBR
18	Italy	ITA	37	United States	USA
19	Japan	JPN			

As we can see from Table 2, the average vaccination rate varies considerably across the OECD members, ranging from the lowest value of 3460.04 in Slovakia to the highest value of 6915.48 in Chile. It can be seen that only five countries, i.e., Slovakia, Mexico, Colombia, Israel, and Latvia, remain in the group of average vaccination rates below 4,000. Moreover, most OECD countries have an average vaccination rate of 4,000-6,000. Seven countries, i.e., Japan, Denmark, Spain, Canada, Portugal, Iceland, and Chile, achieve a vaccination rate above 6,000 on average.

**Table 2:** Average vaccination rate per country.

**Country**	**Average vaccination rates**	**Country**	**Average vaccination rates**
Slovakia	3460.04	Germany	5390.57
Mexico	3583.24	Turkey	5394.63
Colombia	3707.15	Sweden	5681.46
Israel	3876.36	Netherlands	5733.83
Latvia	3912.06	Finland	5826.93
Poland	4038.47	Norway	5923.62
Estonia	4243.03	South Korea	5957.09
United States	4349.13	France	5963.31
Slovenia	4403.50	Belgium	5973.78
Czechia	4582.24	Italy	5980.93
United Kingdom	4777.22	Ireland	5996.18
Greece	4846.25	Japan	6115.22
Costa Rica	4954.55	Denmark	6209.51
Lithuania	4972.85	Spain	6291.72
Switzerland	4993.09	Canada	6366.72
Austria	5070.50	Portugal	6569.92
Luxembourg	5265.96	Iceland	6748.58
Australia	5308.58	Chile	6915.48
New Zealand	5368.95		

*The average vaccination rate is calculated as the mean value of the daily vaccination per million of the population for each panel of OECD member countries*.

Table 3 further elaborates the details of descriptive statistics of average vaccination rates across the OECD countries. It can be seen that across the panel of 37 countries in the OECD region, the mean value of the vaccination rates is 5263.59, which is above the average vaccine rates of 16 countries included in the sample. Furthermore, the standard deviation of the cross-country vaccination rates is 939.704, which indicates a significant amount of volatility. We believe this is due to the fact that the average vaccination rate varies considerably as it ranging from 3460.04 in Slovakia to 6915.48 in Chile.

**Table 3:** Descriptive statistics.

**Variable**	**Obs**	**Mean**	**Std. Dev**.	**Min**	**Max**
Average Vaccination Rate	37	5263.59	939.704	3460.044	6915.485

*The average vaccination rate is calculated as the mean value of the daily vaccination per million of the population for each panel of OECD member countries*.

**Figure 1:**
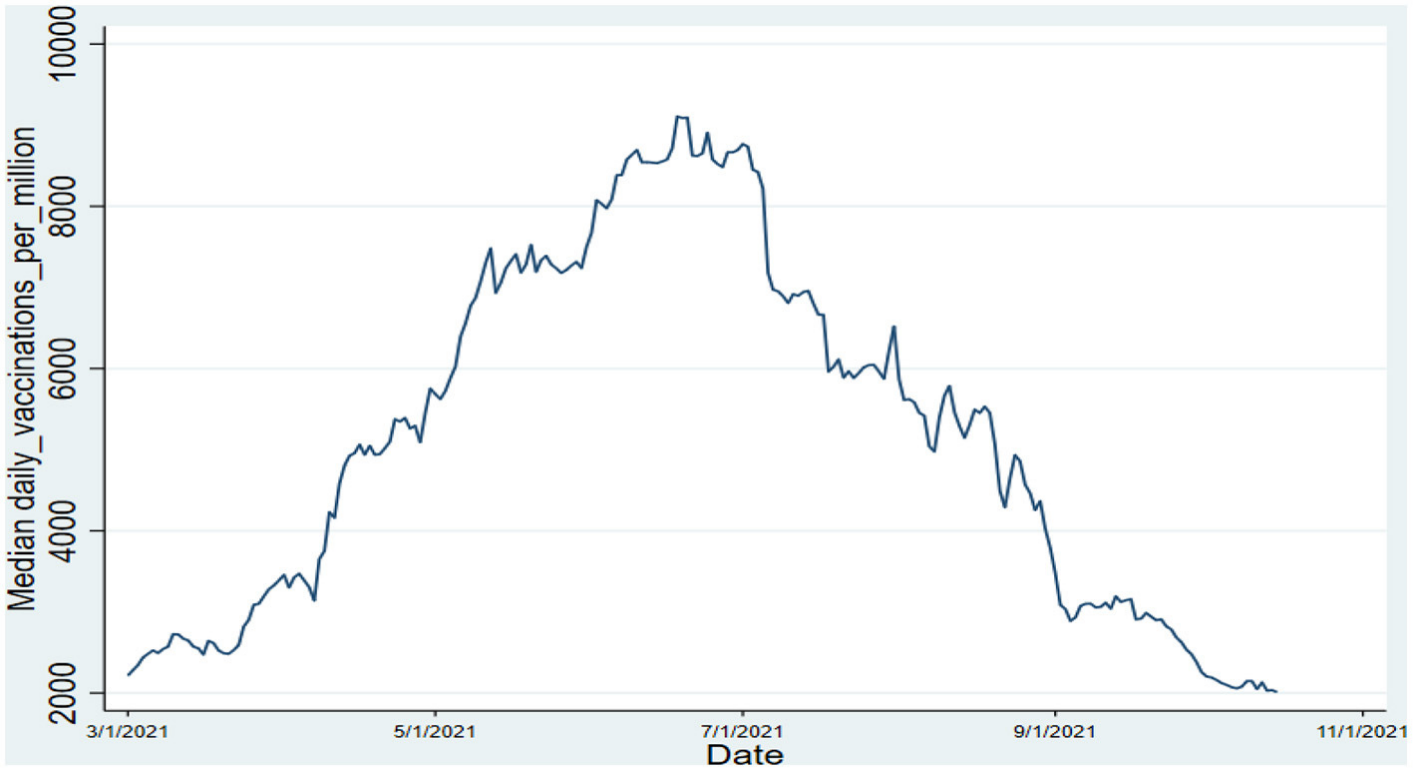
Dynamics of median vaccination rate over time. Source: author's calculations.

Since we are also interested in the time-evolving trend of vaccination rates in the OECD regions, Figure 1 depicts the median value across the panel of 37 OECD member countries during the sample period between March 1, 2021, and October 15, 2021. It is seen that the median vaccination rate increased steadily from the beginning of March and reached a peak over the summer period of 2021. However, it is found that the median vaccination rate started to drop in the second half of 2021 and reverted to below the initial level with a value of slightly above 2000 at the end of the sample period.

### Convergence of the Vaccination Rates in OECD Regions

As described in the methodology section, we employ the algorithm proposed by Phillips and Sul (9, 10) to formally test the convergence pattern and detect potential clustering clubs of OECD member countries. We present the results of convergent and non-convergent countries in Table 4.

**Table 4:** Convergence pattern of vaccination rates in 37 OECD countries.

**Group**	**T-statistic**	**Coefficient**
Full Sample	−12.228	−2.487
Club 1: (9)		
1 5 7 15 17 19 25 31 35	10.486	0.672
Club 2: (11)		
4 6 11 12 18 20 21 23 33 34 37	−0.213	−0.007
Club 3: (2)		
13 14	−0.274	−0.615
Club 4: (2)		
22 27	−1.255	−2.44
Club 5: (2)		
2 10	−0.917	−0.437
Club 6: (2)		
24 36	0.099	0.249
Club 7: (2)		
8 29	−0.13	−0.241
Not convergent Group 8: (7)		
3 9 16 26 28 30 32	−105.408	−3.977

As shown in the first row of Table 4, the t-statistic of−12.22 is < −1.65, indicating that the null hypothesis of convergence is rejected at the 5% level for the full sample of countries. As we can see from the rest of Table 4, seven convergent clubs are formed between March 1, 2021, and October 15, 2021. More specifically, club one consists of Australia, Chile, Costa Rica, Iceland, Israel, Japan, New Zealand, South Korea, Turkey. The third row of Tables shows that the t-statistic is−0.213, >-1.65, implying that the eleven countries of Canada, Colombia, Finland, France, Italy, Latvia, Lithuania, Mexico, Sweden, Switzerland, and the United States is formed as the second group.

Interestingly, club three to seven is composed of two countries, respectively. In particular, club three includes Germany and Greece, while club four comprises Luxembourg and Poland. Furthermore, Austria and Estonia are in group five, while the Netherlands and the United Kingdom are included in group six. Group seven includes two countries in Central Europe: Czechia and Slovakia. Finally, the t-statistic in the last row of Table 4 is −105.408, which provides empirical evidence that a group of seven countries comprises Belgium, Denmark, Ireland, Norway, Portugal, Slovenia, Spain shows no sign of convergence.

According to the study of Cakmakli et al. (39), worldwide income can be raised by 9 Trillion USD through the acceleration of recovery from the COVID-19 pandemic, with an approximate income increase of 4 Trillion USD contributed to developed economies. Given the vast advantage of the faster progress toward the rollout of vaccination, OECD member countries must collaborate and use their comparative advantages. For instance, the countries in the OECD region that possessed the capacity of vaccine development and manufacturing should cooperate with the OECD member countries with advanced logistics and supply chain systems to allow for an efficient distribution of vaccination. In addition, the existence of non-converging countries might arise from inequitable access to the vaccine, which calls for policy support to tackle this challenge.

We further examine any possible club merging and reports the results in Table 5. The log t regression test for all pairs of adjacent clubs shows that t-statistic is consistently < -1.65 in columns 2 to 8 of Table 5, indicating that the null hypothesis of convergence is rejected at the 5% level. No adjacent subgroups can be merged into a larger club.

**Table 5:** Test of club merging.

**log(t)**	**Club1+2**	**Club2+3**	**Club3+4**	**Club4+5**	**Club5+6**	**Club6+7**	**Club7+G**~**8**
Coefficient	−1.003	−0.455	−3.51	−2.281	−1.903	−0.869	−4.079
T-statistic	−15.422	−28.842	−6.796	−23.029	−21.697	−4.264	−24.955

## Conclusion

Vaccines are essential to create a more resilient economic growth model. Ending the COVID-19 pandemic requires a more coordinated, effective, and equitable distribution of vaccines across the countries. Therefore, governments are in a race to increase the vaccination rates of the population. Given this backdrop, this paper analyses the convergence patterns of daily vaccinations per million data in 37 OECD countries from March 1, 2021, to October 15, 2021. The paper examines the stochastic properties of the vaccination rates. The results indicate a significant seven convergence clubs. At the same time, seven countries demonstrate non-convergent characteristics, which raises questions about ineffective vaccine balance.

Significant factors are contributing to countries' ineffective vaccine balance: myths, religion, and even skepticism and mistrust issues. Suspicion and fear about vaccination are widespread, particularly among certain “marginalized” groups in developed countries. In developing countries such as Asia and Africa, vaccine distrust is sometimes associated with “Western plot” ideas, which assert that vaccines are used to sterilize or infect non-Western cultures. Therefore, the OECD governments must provide region-specific policy and facilitate a smooth vaccine rolling out to ensure speedy vaccination progress. Future papers can focus on the determinants of the vaccination rates in OECD countries.

## Data Availability Statement

Publicly available datasets were analyzed in this study. This data can be found here: https://ourworldindata.org/covid-vaccinations.

## Author Contributions

WX: writing—review and editing and funding acquisition. CL: supervision, methodology, and writing—review and editing. DZ: software and methodology. OO: writing—writing and editing. All authors contributed to the article and approved the submitted version.

## Conflict of Interest

The authors declare that the research was conducted in the absence of any commercial or financial relationships that could be construed as a potential conflict of interest.

## Publisher's Note

All claims expressed in this article are solely those of the authors and do not necessarily represent those of their affiliated organizations, or those of the publisher, the editors and the reviewers. Any product that may be evaluated in this article, or claim that may be made by its manufacturer, is not guaranteed or endorsed by the publisher.

## Footnotes

^1^The two lowest are 17% in Russia,19% in Japan, and the highest are 72% in Brazil and 67% in the United Kingdom.

^2^The data sources and types of vaccines used in each member country in OECD regions can be from https://ourworldindata.org/COVID-vaccinations.

^3^Hungary is excluded since there are many gaps in the data.
